# Diabetic dermopathy (“shin spots”) and diabetic bullae (“bullosis diabeticorum”) at the same patient

**DOI:** 10.12669/pjms.315.7521

**Published:** 2015

**Authors:** Piotr Brzezinski, Anca E Chiriac, Tudor Pinteala, Liliana Foia, Anca Chiriac

**Affiliations:** 1 Dr. Piotr Brzezinski, MD, PhD. Department of Dermatology, 6^th^ Military Support Unit, Slupsk, Poland; 2 Anca E Chiriac, MD, PhD. Student, University of Medicine and Pharmacy “Gr. T. Popa”, Iasi, Romania; 3 Tudor Pinteala, MD. Student, University of Medicine and Pharmacy “Gr. T. Popa”, Iasi, Romania; 4 Prof. Liliana Foia, MD, PhD. University of Medicine and Pharmacy “Gr. T. Popa”, Iasi, Romania; 5 Prof. Anca Chiriac, MD, PhD. Department of Dermatology, University Apollonia, Nicolina Medical Center, “P.Poni” Research Institute, Iasi, Romania

**Keywords:** Diabetes mellitus, Microvascular, Insulin, Metabolic, Dermatosis

## Abstract

We present a diabetic patient with associated two diabetic dermatoses: diabetic dermopathy (“shin spots”) and diabetic bullae. A 34-year-old man, with long history of diabetes mellitus, hypertension, and moderate obesity presented to Dermatology Unit for diagnosis of his skin lesions. On clinical examination multiple, light brown, irregular patches, with atrophic scars and crusts over large bullae were observed on the anterior aspect of both legs.

A 34-year-old man, with long history of diabetes mellitus, hypertension, and moderate obesity presented to Dermatology Unit for diagnosis of his skin lesions. On clinical examination multiple, light brown, irregular patches, with atrophic scars and crusts over large bullae were observed on the anterior aspect of both legs (Fig.1). The skin lesions were totally asymptomatic, no previous trauma reported by the patient, no drug intake apart from insulin. Thepatient reported the appearance of lesions in crops, over the last one year, despite good control of metabolic status.

**Fig.1:**
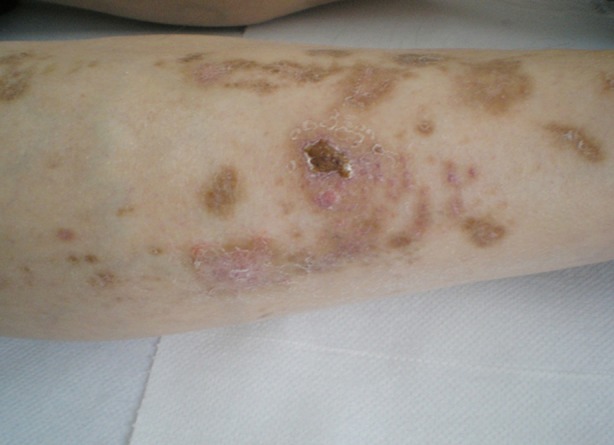
Diabetic dermopathy and crusts over large bullae on bilateral pretibial areas.

His glycemic control was good with glycosylated hemoglobin (HbA1c) under 6-6.8%, he had no neuropathy or vascular associated pathology. For excluding chronic venous insufficiency clinically expressed by edema, pruritus stasis dermatitis, hair loss, lipodermatosclerosis with or without venous ulceration, duplex ultrasonography was performed and found to be within normal limits.

A diagnosis of diabetic dermopathy (“shin spots”) and diabetic bullae in the same patient was taken into consideration. Antibiotics administered orally, careful hygiene, topical steroids class III and emollients were recommended and a close follow-up of the patient was ensured for the next 6 months.

A recent report, evaluating the diabetic complications, revealed astonishing data: 38.0% of patients enrolled in the study had neuropathy, 23.3% had nephropathy, 22.9% had retinopathy and 79.2% skin involvement.^1^

The prevalence of cutaneous manifestations are (is) reported equally in type 1 DM and type 2 DM, but statistically type 2 DM patients are prone to cutaneous infections, while type 1 DM patients are more susceptible to autoimmune cutaneous disorders.

Among cutaneous manifestations of diabetes mellitus, diabetic dermopathy, also named “shin spots” and diabetic bullae (‘bullosisdiabeticorum”) are considered markers of diabetes or skin lesions with strong association.^2^

Diabetic dermopathy is described in variable percentage of patients: 36% of the patients or 7–70% of the diabetics, more often in men over 50 years and it is related pathogenic ally with diabetic microangiopathy.^3^,^4^ Bullosis diabeticorumis rarely reported (0.4%) patients although it is admitted as a marker of diabetes mellitus.^4^

Peculiarities of present case were young diabetic patient, type I diabetes mellitus, good glycemic control, no comorbidities, two cutaneous markers‘diabetic dermopathy and diabetic bulla’ present at the same time.
